# One-Year Follow-Up of a Randomized Controlled Trial Piloting a Mindfulness-Based Group Intervention for Adolescent Insulin Resistance

**DOI:** 10.3389/fpsyg.2019.01040

**Published:** 2019-05-08

**Authors:** Lauren B. Shomaker, Bernadette Pivarunas, Shelly K. Annameier, Lauren Gulley, Jordan Quaglia, Kirk Warren Brown, Patricia Broderick, Christopher Bell

**Affiliations:** ^1^Department of Human Development and Family Studies, Colorado State University, Fort Collins, CO, United States; ^2^Department of Community and Behavioral Health, Colorado School of Public Health, Aurora, CO, United States; ^3^Section of Endocrinology, Department of Pediatrics, School of Medicine, University of Colorado, Aurora, CO, United States; ^4^Department of Contemplative Psychology, Naropa University, Boulder, CO, United States; ^5^Department of Psychology, Virginia Commonwealth University, Richmond, VA, United States; ^6^Edna Bennett Pierce Prevention Research Center, The Pennsylvania State University, University Park, PA, United States; ^7^Department of Health and Exercise Science, Colorado State University, Fort Collins, CO, United States

**Keywords:** mindfulness, cognitive-behavioral therapy, depression, insulin resistance, type 2 diabetes, adolescents

## Abstract

**Introduction:**

To explore if a brief mindfulness-based intervention (MBI) leads to sustained, improved clinical outcomes in adolescents at-risk for type 2 diabetes (T2D).

**Methods:**

Participants were 12–17y girls with overweight/obesity, elevated depression symptoms, and T2D family history participating in a randomized, controlled pilot trial of a six-session MBI vs. cognitive-behavioral therapy (CBT) group. At baseline and 1-year, mindfulness, depression, insulin resistance (IR), and body composition were assessed with validated instruments.

**Results:**

One-year retention was 71% (*n* = 12) in MBI; 81% (*n* = 13) in CBT. At 1-year, depression decreased (Cohen’s *d* = 0.68) and IR decreased (*d* = 0.73) in adolescents randomized to MBI compared to those in CBT. There were no significant between-condition differences in mindfulness, adiposity, or BMI.

**Discussion:**

One-year outcomes from this randomized, controlled pilot trial suggest that brief MBI may reduce depression and IR in at-risk adolescents. Replication and exploration of mechanisms within the context of a larger clinical trial are necessary.

**Clinical Trial Registration:**

www.ClinicalTrials.gov, identifier NCT02218138.

## Introduction

Mindfulness has been described in clinical contexts as paying attention in a purposeful way to the present moment, without judgment (Kabat-Zinn, 1994). In adults, mindfulness-based interventions (MBIs) have been utilized for managing common, chronic health conditions like type 2 diabetes (T2D). Randomized controlled trials in adults with diabetes show that MBIs have small-to-moderate effects for decreasing depression and heterogeneous effects for glycemic control (Abbott et al., 2014). MBIs are designed to address depression by increasing frequency of mindful states, thereby providing individuals with more effective ways of coping with stressors that accompany major health conditions.

Learning mindfulness-based coping skills during adolescence has potential to alter progression of metabolic abnormalities. In particular, adolescence is a sensitive period for insulin resistance (IR) that accompanies puberty. Pubertal IR triggers a trajectory toward worsening IR and eventual T2D in vulnerable youth (Kelsey and Zeitler, 2016). Adolescents at-risk for T2D are disproportionately female, from historically marginalized race/ethnicities, and frequently experience depression (Walders-Abramson et al., 2013). Depression symptoms are associated with greater IR in adolescents, predict worsening IR over time, and relate to T2D onset in young adulthood (Shomaker et al., 2011; Suglia et al., 2016). Depression is hypothesized to affect IR, independent of energy balance, through stress-related behavior (e.g., emotional-eating) and physiology (e.g., hypercortisolism). Therefore, intervening to increase mindfulness and decrease depression in adolescents at-risk for T2D is anticipated to ameliorate IR. MBIs may have salutary effects on IR through a number of mechanisms, including decreases in depression and increases in effective self-regulation of attention, stress, emotion, and behaviors important for IR, such as emotional eating (Lyons and Zelazo, 2011).

We conducted a randomized, controlled trial to pilot an MBI in adolescent girls at-risk for T2D with elevated depression symptoms. We had positive findings for the primary outcome of feasibility/acceptability (Shomaker et al., 2017). In secondary analyses, we found that adolescents in MBI had larger post-treatment decreases in depression and IR and larger 6-month decreases in depression, compared to cognitive-behavioral therapy (CBT; Shomaker et al., 2017).

The objective of this brief report was to explore 1-year outcomes. We hypothesized that adolescents randomized to MBI would have lower depression and IR at 1-year than CBT participants, based upon our initial findings and the notion that MBI may be uniquely suited for the high-stress arousal and psychosocial context of adolescents at-risk for T2D (Shomaker et al., 2017). In contrast to CBT depression interventions that focus on restructuring negative thoughts and increasing positive behaviors, MBI centers on cultivating self-regulation through present-focused, non-judgmental attention to one’s body, emotions, and thoughts. The current analysis directly addresses shortcomings of existing literature by relying on a randomized controlled design, using an active comparator, and conducting long-term follow-up (Van Dam et al., 2017).

## Methods

### Participants and Procedures

Adolescents were recruited for a prevention of T2D pilot trial, conducted at an academic setting in the western region of the United States between January 2014–August 2016 (ClinicalTrials.gov: NCT02218138). Participants were girls, mean ± SD_age_ 14.99 ± 1.69y (Range 12–17y) of all racial/ethnic backgrounds (70% non-Hispanic White, 21% Hispanic, 9% American Indian) predisposed to elevated IR by overweight/obesity (BMI ≥ 85th percentile) and T2D family history. The Institutional Review Board of Colorado State University approved all procedures. Youth had elevated depression, based on a total score ≥ 16 on the Center for Epidemiologic Studies-Depression Scale (CES-D_totalscore_ 25.27 ± 6.63). Baseline exclusion criteria were pregnancy, major depressive disorder or diagnosis warranting treatment, major medical problem (e.g., T2D), medication affecting mood/insulin, or psychotherapy. Parental guardians provided written informed consent, and adolescents gave written assent, after having the study described to them in detail.

Following an appointment to screen eligibility and collect baseline assessments, adolescents were randomized to a six-session MBI group (Learning to BREATHE; *n* = 17) or six-session CBT depression prevention group (Blues Program; *n* = 15) (Shomaker et al., 2017). The randomization sequence was generated by an electronic program using permuted blocks and stratified by age and race/ethnicity. Interventions were matched for time and attention. Sessions were co-facilitated by a clinical psychologist and graduate student in psychology or marriage and family therapy. Phlebotomists were blinded to allocation; trained research associates or graduate research assistants served as assessors of psychosocial characteristics and body measurements, and they were not consistently blinded.

### Measures

For the current brief report, measures collected at baseline and 1-year were evaluated. The CES-D measured depression symptoms and the Mindful Attention Awareness Scale (MAAS) assessed basic dispositional mindfulness. IR was estimated with homeostasis model assessment of IR (HOMA-IR), a surrogate with good convergent validity with hyperinsulinemic euglycemic clamp-derived measures (George et al., 2011). Adiposity was measured as percentage total fat-mass from dual-energy x-ray absorptiometry. Tanner-breast stage was self-reported. BMI-metrics were determined from fasting weight/height according to Centers for Disease Control and Prevention 2000 standards.

### Analytic Plan

A pilot sample size of *n* = 15–25 per arm is recommended for informing a main trial in which small-to-moderate standardized effects are anticipated (Whitehead et al., 2016). All variables approximated a normal distribution. Using IBM SPSS Statistics 25, EM (expectation–maximation) was used to impute missing data. Univariate ANCOVA with the intent-to-treat sample was used to evaluate condition (MBI vs. CBT) as a predictor of baseline-to-1-year change in depression and IR. Covariates were race/ethnicity, baseline level of each outcome, age, weight status (overweight BMI 85th–94th percentile vs. obesity BMI ≥ 95th percentile), puberty (Tanner 5 reported breast development vs. Tanner 3–4), adiposity, and 1-year adiposity change. ANCOVA also was used to describe between-condition and within-condition differences in baseline-to-1-year change in dispositional mindfulness, BMI indices, and adiposity. In addition to statistical significance (*p* < 0.05), we estimated effect size with Cohen’s *d* (small: 0.2, medium: 0.5, large ≥ 0.8).

## Results

Descriptive information on baseline and 1-year sample characteristics is provided in Supplementary Table 1. One-year retention was 71% (*n* = 12) in MBI; 81% (*n* = 13) in CBT (*p* = 0.48). Baseline characteristics were not significantly related to retention.

**FIGURE 1:**
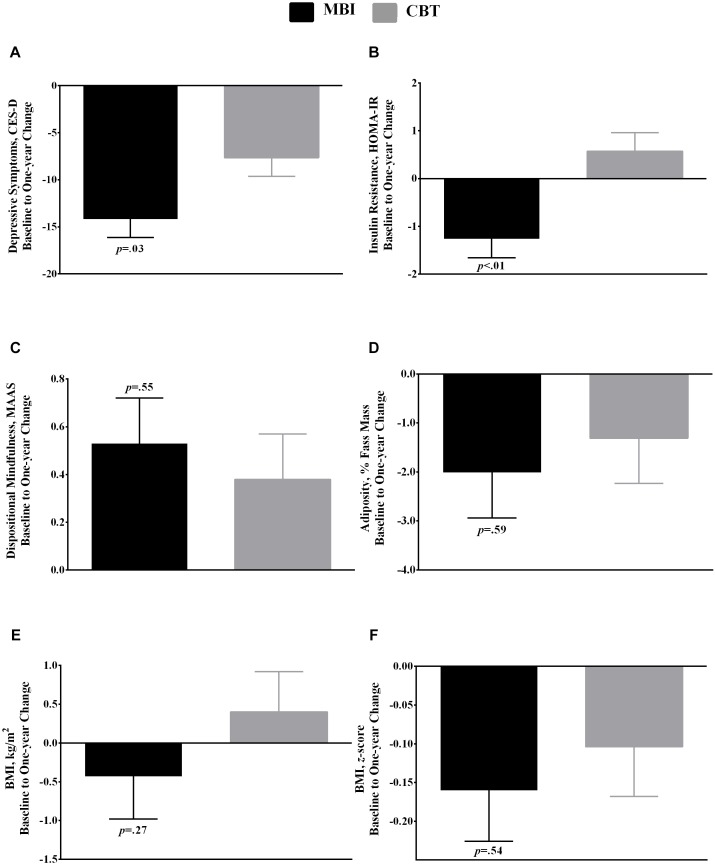
Baseline to 1-year change (Mean, SE) in **(A)** depression symptoms, **(B)** homeostasis model assessment of insulin resistance (HOMA-IR), **(C)** dispositional mindfulness, **(D)** adiposity (%), **(E)** body mass index (BMI; k/gm^2^), and **(F)** BMI *z*-score by condition: MBI (mindfulness-based group intervention; *n* = 17) vs. CBT (cognitive-behavioral therapy; *n* = 16) group intervention. *P*-values refer to the between-group difference in baseline to 1-year change, derived from univariate analyses of covariance (ANCOVA) using the intent-to-treat sample with EM (expectation–maximation) to handle missing data. Models a, b, and c accounted for race/ethnicity, baseline level of the outcome, baseline age, baseline weight status (overweight BMI 85th–94th percentile vs. obesity BMI ≥ 95th percentile), baseline puberty (Tanner 5 reported breast development vs. Tanner 3–4), baseline adiposity, and 1-year change in adiposity. Models d and e accounted for race/ethnicity, baseline level of the outcome, baseline age, baseline weight status, and baseline puberty.

Table 1 summarizes baseline-to-1-year changes by condition, accounting for covariates. Depression decreased from baseline-to-1-year within MBI and CBT, with a greater decrease in MBI (*d* = −0.68, *p* = 0.03; Figure 1). Adolescents in MBI decreased baseline-to-1-year IR compared to stable IR in CBT, with a moderate, between-condition effect size(*d* = 0.73, *p* < 0.01).

**Table 1:** Summary of univariate analyses of covariance (ANCOVA) predicting change in outcome from group condition.

Baseline to 1-year change	MBI^‡^	CBT^‡^	Between-group effect^‡^	*p*-value	Cohen’s *d* effect size
Depression symptoms	−14.17 [−18.23, −10.12]	−7.65 [−11.67, −3.63]	−6.52 [−12.22, −0.82]	0.03	0.68
Insulin resistance	−1.26 [−2.07, −0.44]	0.57 [−0.22, 1.36]	−1.83 [−2.92, −0.73]	<0.01	0.73
Dispositional mindfulness	0.53 [0.14, 0.93]	0.38 [−0.01, 0.77]	0.16 [−0.38, 0.69]	0.55	0.17
Body fat (%)	−2.01 [−3.93, −0.10]	−1.31 [−3.21, 0.58]	−0.70 [−3.34, 1.94]	0.59	0.18
BMI (kg/m^2^)	−0.43 [−1.57, 0.71]	0.40 [−0.67, 1.48]	−0.83 [−2.36, 0.70]	0.27	0.44
BMI, *z*-score	−0.16 [−0.30, −0.03]	−0.10 [−0.24, 0.03]	−0.06 [−0.24, 0.13]	0.54	0.25

*^‡^Mean [95% CI]. MBI, mindfulness-based intervention, n = 17. CBT, cognitive-behavioral therapy, n = 16. BMI, body mass index. Analyses predicting mindfulness, depression, and insulin resistance accounted for race/ethnicity, baseline level of the outcome, baseline age, baseline weight status (overweight BMI 85th–94th percentile vs. obesity BMI ≥ 95th percentile), baseline puberty (Tanner 5 reported breast development vs. Tanner 3–4). Analyses predicting body fat and BMI indices accounted for race/ethnicity, baseline level of the outcome, baseline age, baseline weight status, and baseline puberty.*

There were no other between-condition differences. Mindfulness increased from baseline-to-1-year within MBI, with no within-group change in CBT (*d*_between–group_ = 0.17, *p* = 0.55). Adiposity decreased within MBI, with no change within CBT (*d*_between–group_ = 0.18, *p* = 0.59). Likewise, BMI *z*-score decreased within MBI, with no change within CBT (*d*_between–group_ = 0.25, *p* = 0.54). BMI raw-score change was stable within both conditions (*d*_between–group_ = 0.44, *p* = 0.27).

## Discussion

Consistent with previous findings (Shomaker et al., 2017), adolescents at-risk for T2D with elevated depression had greater decreases in depression symptoms 1-year following a six-session MBI, as compared to adolescents who were randomized to CBT. Yet, adolescents in both conditions significantly decreased depression symptoms. Additionally, even after accounting for change in adiposity, we observed a moderate between-condition effect for MBI in reducing 1-year IR, as compared to stable IR in CBT. To our knowledge, this is the first randomized controlled trial to pilot a comparison of MBI and CBT in adolescents. In adults with vascular disease, MBIs have consistently shown greater decreases in depression symptoms as compared to a control condition (Abbott et al., 2014), but equivocal effects for decreasing depression as compared to CBT (Tovote et al., 2015).

In this pilot study, dispositional mindfulness increased at 1-year within MBI only, but the between-condition difference was a non-significant, small effect relative to CBT. One possibility is that by more effectively decreasing depression, MBI improves underlying behavioral and/or physiological factors that affect IR such as sleep, physical activity, eating behavior, and stress arousal. A significant limitation is the pilot nature of the current study; the small sample size warrants caution regarding interpretation of effect sizes (Kraemer et al., 2006). Provided replication with a larger sample, the current findings suggest there may be promise for relatively brief MBI approaches to lessen T2D risk in adolescents with depression. Differentiating mechanisms of MBI vs. CBT in adolescents at-risk for T2D is an important step for future research. Pinpointing mechanisms would help to refine theoretical models and ultimately, aid in developing scalable integrative health interventions for this group of at-risk adolescents.

## Ethics Statement

This study was carried out in accordance with the recommendations of American Psychological Association with written informed consent from all subjects. All subjects gave written informed consent in accordance with the Declaration of Helsinki. The protocol was approved by the Institutional Review Board of Colorado State University.

## Author Contributions

LS conceived the research design, obtained funding for the study, facilitated the interventions, oversaw data collection, conducted the data analysis, and drafted the manuscript. BP facilitated the interventions, collected the data, and edited the manuscript. SA cleaned the data, contributed to the interpretation of results, and edited the manuscript. LG contributed to the interpretations of results and edited the manuscript. JQ assisted in designing the study, contributed to the data collection, interpreted the results, and edited the manuscript. KB assisted in designing and obtaining funding for the study, interpreted the results, and edited the manuscript. PB designed the intervention, trained and supervised the facilitators, and edited the manuscript. CB assisted in designing and obtaining funding for the study, conducted the statistical analysis, interpreted the results, and edited the manuscript.

## Conflict of Interest Statement

PB, author of Learning to BREATHE, receives royalty fees from New Harbinger Publications. The remaining authors declare that the research was conducted in the absence of any commercial or financial relationships that could be construed as a potential conflict of interest.

## References

[B1] Abbott R. A. Whear R. Rodgers L. R. Bethel A. Thompson Coon J. Kuyken W. (2014). Effectiveness of mindfulness-based stress reduction and mindfulness based cognitive therapy in vascular disease: a systematic review and meta-analysis of randomised controlled trials. *J. Psychosom. Res.* 76 341–351. 10.1016/j.jpsychores.2014.02.012 24745774

[B2] George L. Bacha F. Lee S. Tfayli H. Andreatta E. Arslanian S. (2011). Surrogate estimates of insulin sensitivity in obese youth along the spectrum of glucose tolerance from normal to prediabetes to diabetes. *J. Clin. Endocrinol. Metab.* 96 2136–2145. 10.1210/jc.2010-2813 21508130 PMC3205514

[B3] Kabat-Zinn J. (1994). *Wherever you go, There you are*. New York, NY: Hyperion.

[B4] Kelsey M. M. Zeitler P. S. (2016). Insulin resistance of puberty. *Curr. Diab. Rep.* 16:64. 10.1007/s11892-016-0751-5 27179965

[B5] Kraemer H. C. Mintz J. Noda A. Tinklenberg J. Yesavage J. A. (2006). Caution regarding the use of pilot studies to guide power calculations for study proposals. *Arch. Gen. Psychiatry* 63 484–489. 10.1001/archpsyc.63.5.484 16651505

[B6] Lyons K. E. Zelazo P. D. (2011). Monitoring, metacognition, and executive function: elucidating the role of self-reflection in the development of self-regulation. *Adv. Child Dev. Behav.* 40 379–412. 10.1016/b978-0-12-386491-8.00010-4 21887967

[B7] Shomaker L. B. Bruggink S. Pivarunas B. Skoranski A. Foss J. Chaffin E. (2017). Pilot randomized controlled trial of a mindfulness-based group intervention in adolescent girls at risk for type 2 diabetes with depressive symptoms. *Compl. Ther. Med.* 32 66–74. 10.1016/j.ctim.2017.04.003 28619307 PMC5705100

[B8] Shomaker L. B. Tanofsky-Kraff M. Stern E. A. Miller R. Zocca J. M. Field S. E. (2011). Longitudinal study of depressive symptoms and progression of insulin resistance in youth at risk for adult obesity. *Diabetes Care* 34 2458–2463. 10.2337/dc11-1131 21911779 PMC3198302

[B9] Suglia S. F. Demmer R. T. Wahi R. Keyes K. M. Koenen K. C. (2016). Depressive symptoms during adolescence and young adulthood and the development of type 2 diabetes mellitus. *Am. J. Epidemiol.* 183 269–276. 10.1093/aje/kwv149 26838597 PMC4753278

[B10] Tovote K. A. Schroevers M. J. Snippe E. Sanderman R. Links T. P. Emmelkamp P. M. (2015). Long-term effects of individual mindfulness-based cognitive therapy and cognitive behavior therapy for depressive symptoms in patients with diabetes: a randomized trial. *Psychother. Psychosom.* 84 186–187. 10.1159/000375453 25832365

[B11] Van Dam N. T. van Vugt M. K. Vago D. R. Schmalzl L. Saron C. D. Olendzki A. (2017). Mind the hype: a critical evaluation and prescriptive agenda for research on mindfulness and meditation. *Perspect. Psychol. Sci.* 13 36–61. 10.1177/1745691617709589 29016274 PMC5758421

[B12] Walders-Abramson N. Nadeau K. J. Kelsey M. M. Schmiege S. J. Ellert S. Cejka A. (2013). Psychological functioning in adolescents with obesity co-morbidities. *Child. Obes.* 9 319–325. 10.1089/chi.2012.0120 23763659 PMC3728721

[B13] Whitehead A. L. Julious S. A. Cooper C. L. Campbell M. J. (2016). Estimating the sample size for a pilot randomised trial to minimise the overall trial sample size for the external pilot and main trial for a continuous outcome variable. *Stat. Methods Med. Res.* 25 1057–1073. 10.1177/0962280215588241 26092476 PMC4876429

