# Supplementary material for: One-Year Follow-Up of a Randomized Controlled Trial Piloting a Mindfulness-Based Group Intervention for Adolescent Insulin Resistance
Source: Front Psychol. 2019 May 8;10:1040. doi: 10.3389/fpsyg.2019.01040 (PMC6517501; doi:10.3389/fpsyg.2019.01040)
Supplement: Supplementary file 1 [file Table_1.pdf]

Supplemental Table 1. Descriptive information about the study sample at baseline and one-year follow-up by group condition

	MBI		CBT	
**Variable**	**Baseline**	**One-year**	**Baseline**	**One-year**
Age, years	15.01 (1.68)	16.17 (1.69)	14.97 (1.75)	16.14 (1.73)
Race/ethnicity				
Non-Hispanic White	*n*=12, 70.6%	--	*n*=11, 68.8%	--
Hispanic	*n*=4, 23.5%	--	*n*=3, 18.8%	--
American Indian	*n*=1, 5.9%	--	*n*=2, 12.5%	--
BMI, kg/m^2	30.48 (5.21)	30.17 (5.61)	29.19 (6.95)	30.11 (8.51)
BMI, z-score	1.81 (.40)	1.70 (.42)	1.61 (.50)	1.59 (.73)
BMI, percentile	95.47 (3.49)	94.38 (5.46)	92.77 (4.89)	91.86 (10.70)
Weight status				
Lean, 5-84%ile	*n*=0, 0%	*n*=1, 5.9%	*n*=0, 0%	*n*=2, 12.5%
Overweight, 85-94%ile	*n*=5, 29.4%	*n*=7, 41.2%	*n*=10, 62.5%	*n*=7, 43.8%
Obesity, ≥95%ile	*n*=12, 70.6%	*n*=9, 52.9%	*n*=6, 37.5%	*n*=7, 43.8%
Body fat, %	44.81 (5.49)	41.53 (6.70)	42.86 (5.10)	43.35 (7.06)
Mindfulness	3.47 (.93)	4.24 (1.11)	3.33 (.83)	3.81 (.94)
Depression symptoms	26.87 (6.01)	12.20 (10.39)	23.30 (6.21)	15.42 (9.10)
Depression status				
Low, CES-D<16	*n*=0, 0%	*n*=12, 70.6%	*n*=0, 0%	*n*=12, 75.0%
Mild elevated, CES-D 16-20	*n*=2, 11.8%	*n*=2, 11.8%	*n*=5, 31.3%	*n*=0, 0%
Moderate elevated, CES-D>20	*n*=15, 88.2%	*n*=3, 17.6%	*n*=11, 68.8%	*n*=4, 25.0%
Insulin resistance, HOMA-IR	2.80 (1.32)	2.24 (1.64)	2.26 (1.34)	2.52 (1.87)
Insulin resistance status				
Normal, HOMA-IR<3.16	*n*=13, 76.5%	*n*=13, 76.5%	*n*=13, 81.3%	*n*=12, 75.0%
Elevated, HOMA-IR≥3.16	*n*=4, 23.5%	*n*=4, 23.5%	*n*=3, 18.7%	*n*=4, 25.0%

Values displayed are Mean (SD) unless otherwise noted. MBI=Mindfulness-based intervention, *n*=17. CBT=cognitive-behavioral therapy, *n*=16. BMI=body mass index. CES-D=Center for Epidemiologic Studies-Depression Scale total score. HOMA-IR=homeostasis model assessment of insulin resistance.
